# A Reliable and Reproducible Model for Assessing the Effect of Different Concentrations of *α*-Solanine on Rat Bone Marrow Mesenchymal Stem Cells

**DOI:** 10.1155/2017/2170306

**Published:** 2017-10-22

**Authors:** Adriana Ordóñez-Vásquez, Lorenza Jaramillo-Gómez, Camilo Duran-Correa, Erandi Escamilla-García, Myriam Angélica De la Garza-Ramos, Fernando Suárez-Obando

**Affiliations:** ^1^Instituto de Genética Humana, Facultad de Medicina, Pontificia Universidad Javeriana, Bogotá, Colombia; ^2^Centro de Investigaciones Odontológicas, Facultad de Odontología, Pontificia Universidad Javeriana, Bogotá, Colombia; ^3^Unidad de Odontología Integral y Especialidades, Centro de Investigación y Desarrollo en Ciencias de la Salud, Facultad de Odontología, Universidad Autónoma de Nuevo León, Mexico

## Abstract

Αlpha-solanine (*α*-solanine) is a glycoalkaloid present in potato* (Solanum tuberosum)*. It has been of particular interest because of its toxicity and potential teratogenic effects that include abnormalities of the central nervous system, such as exencephaly, encephalocele, and anophthalmia. Various types of cell culture have been used as experimental models to determine the effect of *α*-solanine on cell physiology. The morphological changes in the mesenchymal stem cell upon exposure to *α*-solanine have not been established. This study aimed to describe a reliable and reproducible model for assessing the structural changes induced by exposure of mouse bone marrow mesenchymal stem cells (MSCs) to different concentrations of *α*-solanine for 24 h. The results demonstrate that nonlethal concentrations of *α*-solanine (2–6 *μ*M) changed the morphology of the cells, including an increase in the number of nucleoli, suggesting elevated protein synthesis, and the formation of spicules. In addition, treatment with *α*-solanine reduced the number of adherent cells and the formation of colonies in culture. Immunophenotypic characterization and staining of MSCs are proposed as a reproducible method that allows description of cells exposed to the glycoalkaloid, *α*-solanine.

## 1. Introduction

Αlpha-solanine (*α*-solanine) is a glycoalkaloid present in potato* (Solanum tuberosum)* and fruits, such as apple* (Malus domestica)*, cherry* (Prunus avium)*, eggplant* (Solanum melongena)*, and tomato* (Solanum lycopersicum)*. In 1820, Desfosses characterized this glycoalkaloid [1]; since then it has been discovered that the concentration of *α*-solanine is higher in the stems, leaves, husk, and tubers of potato [2]. The substance has been of interest to researchers because of its toxicity and possible teratogenicity in humans. For example, early reports associated *α*-solanine with the deaths of several children in Great Britain in 1899 and with the poisoning of several individuals in Germany in 1922 [3–5]. Outbreaks of *α*-solanine poisoning were also reported throughout the 20th century in several countries [6, 7]. The symptoms of poisoning with this glycoalkaloid (respiratory distress, nausea, vomiting, and diarrhea) are related to the inhibition of acetylcholinesterase [8]; however, the precise mechanism underlying its toxic effects remains under investigation.

Alpha-solanine has been of particular interest because of its toxicity and potential teratogenicity, particularly since 1972 when Renwick, in a controversial hypothesis based on epidemiological investigations, suggested an association between the potato tuber and development of neural tube defects (NTD) [9]. The study demonstrated a significant correlation between human populations that consumed large amounts of* Solanum tuberosum* and the development of NTD [10]. This correlation was of interest in the context of the discovery of the slight mutagenicity of *α*-solanine in vitro, its embryotoxicity in experimental animals [8, 11], and teratogenic effects in several mammals [12–14]. Teratogenic effects include abnormalities of the central nervous system, such as exencephaly, encephalocele, and anophthalmia [15]. The association of *α*-solanine with teratogenic effects in animal models is less significant, given that the estimated average consumption of glycoalkaloids derived from potatoes is 12.75 mg/day (around 0.18 mg/kg body weight). This value is close to one-fifth of the dose that has shown toxicity in humans (1 mg/kg body weight) [8]; however, as *α*-solanine is insoluble, its toxic effects may stem from its capacity to accumulate in the body for up to 24 h after ingestion. Moreover, the specific effects of *α*-solanine on human physiology and cell proliferation remain poorly understood [2]. Hence, the long-term effects and tissue accumulation of this glycoalkaloid are aspects of concern.

Various types of cell culture have been used as experimental models to determine the effect of *α*-solanine on cell physiology. For example, studies of toxicity have been performed using human red blood cells [16], embryonic* Xenopus* cells [17], mouse cardiac cells [18], cancer cell lines, fibroblasts [19, 20], and epithelial cells [21]. In many of these studies, cells have been exposed to concentrations of *α*-solanine ranging from 3 to 60 *μ*M. A concentration of 18.5 *μ*M was nontoxic to human melanoma cells but cytotoxic in fibroblasts [20], concentrations up to 60 *μ*M exhibited low cytotoxicity in rat glioma cells [22], and concentrations up to 50 *μ*M altered the integrity of the epithelial barrier and membrane permeability and inhibited proliferation of epithelial cells [21]. Similar effects have been described in breast cancer and liver cancer cell lines [19]. The observed changes in cultured cells are a consequence of the action of *α*-solanine in the cell membrane lipid bilayer [23]. In addition to inhibiting acetylcholine esterase [24], *α*-solanine interferes substantially with the structure and function of the cell membrane [24, 25]. Although the exact mechanism by which *α*-solanine induces changes in membrane barrier function remains unclear [26], the damage observed induces the exit of ions and proteins from mammalian cells [27].

Recently, the relevance of *α*-solanine has increased because of its possible role as an inhibitor of breast, pancreatic, and esophageal cancers and melanoma [19, 20, 28, 29]. The results of these studies suggest the importance of analysis of morphological and structural changes, such as chromatin condensation and the presence of intracytoplasmic vacuoles, in cells exposed to *α*-solanine, along with phenotypic alterations, such as its effect on cell culture expansion, and the morphology and physiology of stem cells [30–32].

The morphological changes in the mesenchymal stem cell upon exposure to *α*-solanine have not been established. In this context, the possible link between *α*-solanine and teratogenicity (toxicity to undifferentiated cells, such as embryonic cells) and the associated complex phenotypic manifestations, such as NTD, are of interest. Given the lack of knowledge of the precise mechanisms of action of *α*-solanine and the fact that teratogenic effects of this compound in humans have not been ruled out, it is imperative to develop protocols to determine its detailed mechanisms of action at nontoxic concentrations in nondifferentiated, living, adherent cells. This study aimed to describe the structural changes induced by exposure of mouse bone marrow mesenchymal stem cells (MSCs) to different concentrations of *α*-solanine for 24 h.

## 2. Materials and Methods

Bone marrow stem cells were obtained from four healthy Lewis strain rats (LEW/SsNHsd). Lewis rats were selected because these animals have a low prevalence of spontaneous neural tube defects (less than 1 : 1000) [33]. None of the donor rats received previous treatment.

### 2.1. Bone Marrow Extraction and Cell Culture

The Lewis rats were euthanized with CO_2_. Immediately afterward, the skin, muscle tissue, and periosteum were dissected from the femur and tibia to expose the bones, which were separated from each limb, stored in saline solution, and transferred immediately from the animal facility to the laboratory for processing. Using surgical instruments, in a laminar flow cabinet (Labconco, Logic, Class II Type A2) the epiphyses of the bones were broken, loaded with 2 ml of base medium (see Preparation of Cultures and Exposure to *α*-Solanine, below) using a syringe, and their interiors flushed. The eluted volume was collected in 15-ml Eppendorf conical tubes and centrifuged (Thermo Scientific, Heraeus Biofuge Primo R) at 650 ×g for 5 min at 4°C. The supernatant was discarded, the pellet was resuspended in 1 ml of base medium, and the total number of cells per ml was determined. Cell counting was performed using the trypan blue exclusion test for cell viability, with 0.4% trypan blue (Sigma, T-8154) in a Neubauer chamber. Cells were seeded in Corning® cell culture flasks with 25 cm^2^ surface area (Corning, CLS430168) at a concentration of 5 × 10^5^ cells per flask and maintained at 37°C with 5% CO_2_ in an incubator (Thermo Scientific, Series 8000 WJ). Culture medium was replaced every three days until the cells reached 90% confluence; this procedure established the primary culture (PC0).

### 2.2. Purification of CD45^+^ Cells and Population Subculture

PC0 cells were detached using 0.25% trypsin and 0.02% EDTA solution (Sigma, T-4049) to form a cell suspension. Cells were then collected by centrifugation at 650 ×g for 5 min at 4°C. The cells were counted, and the CD45^+^ fraction was separated by incubation with an anti-CD45 antibody (BD Biosciences, 554878) for 15 min at 4°C, using 10 *μ*l of anti-CD45 antibody per 1 × 10^6^ cells, washed with a 1 : 20 solution of MACS BSA Stock Solution (MACS Miltenyi Biotec, 130-048-701) in MACS Buffer (AutoMACS™, 130-091-22), then incubated with a secondary Anti-FITC antibody Microbeads (MACS Miltenyi Biotec, 130-048-701) coupled to magnetic beads (Miltenyi Biotech, Germany) for 15 min at 4°C. The suspension was applied to a magnetic separation column (Miltenyi Biotec, LS-MACS Columns), the labeled cells bound to the column, the unlabeled cell fraction, containing the CD45^−^ cell fraction, was eluted and subcultured in base medium, representing the first passage (P1). When P1 cells reached 90% confluency, they were disaggregated, collected by centrifugation, counted, and successively subcultured until they reached the third passage (P3), and then they were used for experiments.

### 2.3. Characterization of Cultured Cells

The immunophenotypic characteristics of the cell cultures at P3 were determined by flow cytometry [34], using specific antibodies coupled with fluorochromes PE, APC, and FITC as follows: CD45-PE (554878, DB Biosciences), CD29-Biot/Strep-APC (555004, DB Biosciences), CD90-FITC (130-094-527, Miltenyi Biotec), CD71 = PE (554891, BD Biosciences), and CD106-PE (559229, BD Biosciences). Cells experimentally exposed to *α*-solanine expressed high levels of the CD90, CD29, CD71, and CD106 stem cell markers. The undifferentiated status of the cells was verified by inducing them to differentiate into an osteogenic lineage, according to standardized laboratory procedures (unpublished results).

### 2.4. Preparation of Cultures and Exposure to *α*-Solanine

The *α*-solanine glycoalkaloid (C_45_H_73_NO_15_, solatunin; *α*-solanine), a trisaccharide consisting of glucose, galactose, rhamnose, and a solanidine aglycone ring (purity > 95%, Santa Cruz Biotechnology SC-252340), was used. Alpha-solanine was solubilized with 0.45% NaCl (Sigma-Aldrich S7653), 0.25% acetic acid (CH_3_COOH, Sigma-Aldrich A9967), and 0.2% dimethyl sulfoxide (Sigma-Aldrich, D2650). MSCs exposed to *α*-solanine were cultured in Corning cell culture flasks, with 25 cm^2^ surface area (Corning CLS430168), in *α*-MEM culture medium (Gibco 12000-014). The medium was supplemented with 10% of fetal bovine serum (Gibco 16000-044), 1% penicillin and streptomycin (Gibco 15240-062), an antimycotic (amphotericin B), 1% GlutaMAX-I (Gibco 35050-061), and 2.2 g sodium bicarbonate (NaHCO_3_, Merck 106329). This *α*-MEM medium was considered base medium for cell culture.

When rat MSC P3 cultures, reached 95% confluence, they were separated into 4 cell culture flasks, marking four of them as experimental groups (A, B, C, and D); the repeated experiments were labeled (A^*∗*^, B^*∗*^, C^*∗*^, and D^*∗*^), and the flasks were subjected to the following treatments: A, A^*∗*^: no exposure to the glycoalkaloid (negative control); B, B^*∗*^: exposure to 2 *μ*M of *α*-solanine; C, C^*∗*^: exposure to 4 *μ*M of *α*-solanine; and D, D^*∗*^: exposure to 6 *μ*M of *α*-solanine. Human fibroblasts exposed to *α*-solanine (18.5 *μ*M) for 24 h [20] were used as positive controls for cytotoxic characterization. Cells were exposed to *α*-solanine at the indicated concentrations for 24 h at 37°C and 5% CO_2_. After treatment, 100 cells were manually counted using inverted microscopy (60x) and a mechanical counter. The number of adhered and nonadherent cells and their phenotypic characteristics were recorded for each of the exposed cultures.

### 2.5. Cell Culture Staining

Cells from each experimental group (A, B, C, and D) were washed with phosphate-buffered saline, pH 7.4 (Sigma P3813), and stained directly in culture with 30 *μ*l of Chinese ink (Pelikan #17 Schwarz Black) (ref. 70306 4340503), following the protocol of Ordóñez Vásquez et al. [35]. After staining the cells, their qualitative (morphological description) and quantitative characteristics, including the number of adhered and nonadhered cells, were assessed by optical microscopy. Among adherent cells, the number of cell colonies and the number of cells forming the colonies were determined. Experiments were performed in duplicate.

### 2.6. Statistical Analysis

To evaluate the significance of differences between the proportions of adhered cells in the treatment groups, a multiproportion comparison test (Marascuilo procedure) with Monte Carlo simulation was used (*α* = 0.05). For comparisons of the numbers of cell colonies and the number of cells in each colony among treatment groups, the Kruskal-Wallis test was used (*α* = 0.05).

## 3. Results

### 3.1. Structural Changes in MSCs Exposed to *α*-Solanine

**Figure 1:**
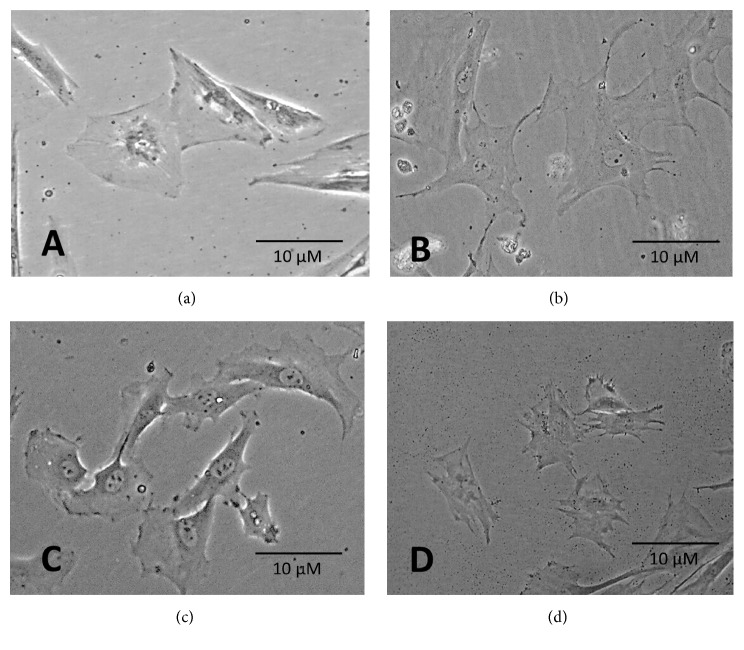
Bone marrow mesenchymal stem cells in culture stained with China ink after 24 hours' exposure to *α*-solanine. (a) No exposure to the glycoalkaloid (negative control); (b) exposure to 2 *μ*M of *α*-solanine; (c) exposure to 4 *μ*M of *α*-solanine; and (d) exposure to 6 *μ*M of *α*-solanine.

Stem cells are typically small, long, and narrow, containing a large round nucleus surrounded by fine chromatin particles that delimit within it one or two rounds and prominent nucleoli. Fibroblastoid cells are classically heterogeneous in appearance and can be elongated or pyramidal, with an even cytoplasmic to nucleus ratio (Figure 1(a)). Figures 1(b)–1(d) show the morphological changes observed in living rat MSCs exposed to *α*-solanine at three nonlethal concentrations for 24 h.

Structural changes first became evident after exposure to 2 *μ*M *α*-solanine and were directly proportional to the concentration used. On treatment with 2 *μ*M *α*-solanine, the nucleus remained slightly elongated and became less heterogeneous, with increased perinuclear cytoplasmic density (Figure 1(b)). Cells exposed to 4 *μ*M of *α*-solanine lost their characteristic morphology, and the nuclei become less compact and larger, presumably reflecting increased protein synthesis. Cells treated with *α*-solanine exhibited signs of distress, including changes in volume, increased cytoplasmic condensation, changes in the proportion of cells occupied by nuclei, and development of elongated spicules (Figure 1(c)). Although no significant changes in cell size are observed at 2 *μ*M and 4 *μ*M exposition, there is a significant decrease in cytoplasmic volume in cells exposed to 6 *μ*M of *α*-solanine, presumably secondary to condensation and progressive cytoplasmic retraction. Small cells, condensed chromatin, with many short spicules, and white spaces that show cytoplasmic vacuoles and cell isolation are all evident; these morphological changes suggest an early apoptosis process. (Figure 1(d))

**Figure 2:**
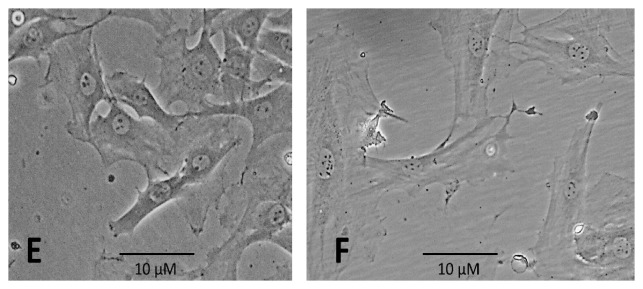
Nucleoli images of bone marrow mesenchymal stem cells in culture stained with China ink after 24 hours' exposure to *α*-solanine. (E) Exposure to 2 *μ*M of *α*-solanine, 1 to 3 regular nucleoli. (F) Exposure to 4 *μ*M of *α*-solanine, 5 to 7 irregular nucleoli.

In groups exposed to higher concentrations of *α*-solanine, we observed differences in the shape of nucleoli. There were no differences in the cell morphology between the control group and the MSC group exposed to 2 *μ*M *α*-solanine; cells contained 1–3 rounded, distinct, and dark nucleoli (Figure 2(E)).

In contrast, MSC cultures exposed to 4 *μ*M of *α*-solanine contained cells with 3–6 nucleoli, all of which were irregular and very small (Figure 2(F)). In MSCs exposed to 6 *μ*M *α*-solanine, no nucleoli-like structures were identified. Characterization of cytoplasmatic extensions/gripping spicules indicated that they appeared to respond directly to increasing *α*-solanine concentration. The number of extensions per cell increased proportionally to glycoalkaloid concentration.

### 3.2. Cell Adhesion at Different Concentrations of *α*-Solanine

**Figure 3:**
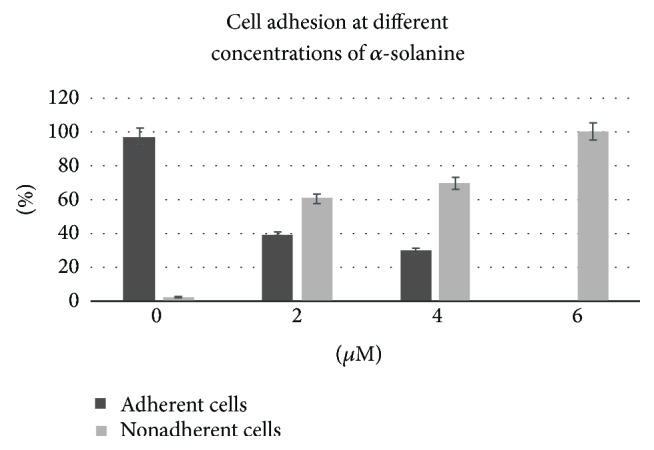
Change of adherent and nonadherent cells proportion as the *α*-solanine concentration increases. The repeated experiments showed the same results (see supplementary data).

Quantification of the total number of adherent cells indicated significant differences among the treatment groups (Figure 3). As the concentration of *α*-solanine increased, the number of adherent cells decreased, reaching zero at the maximum treatment concentration (*p* < 0.0001) (see supplementary material regarding the Marascuilo procedure and repeated experiment results, available online at https://doi.org/10.1155/2017/2170306).

### 3.3. Influence of *α*-Solanine Concentration on Cell Colony Formation

At higher concentrations of *α*-solanine, the number of cell colonies decreased (Kruskal-Wallis test, *X*^2^ = 12.352,   *p* = 0.0061). Moreover, as the concentration increased, the number of cells per colony decreased (Kruskal-Wallis test, *X*^2^ = 21.5, *p* = 0.0001) (Table 1).

## 4. Discussion

Qualitative morphological analysis of MSCs exposed to different concentrations of *α*-solanine was performed, demonstrating dose-dependent phenotypic changes, in cell shape, size, development of cytoplasmic extensions, and adhesion. Under control conditions, MSCs dispersed in culture are small, well-defined cells with large round nuclei, prominent nucleoli, and an adjacent extracellular matrix formed by reticular fibrils [36]. The changes in size, shape, and number of nucleoli observed in this study were associated with structural changes secondary to the accumulation of *α*-solanine in the cytoplasm and within intracellular vacuoles, leading to inhibition of cholinesterases and sodium-potassium ATPase pumps [22]. The observed changes in MSC shape were consistent with previous reports of cellular responses to this glycoalkaloid; for example, exposure of toad embryonic cells to *α*-solanine led to alteration of membrane potential, generating both damage to lipid membrane structure and modification of ion transport channels [37, 38].

The observed changes in cell adhesion, indicating a reduced capacity of cells to adhere to the culture flask surface, as well as reduced cell colony formation, confirm that the membrane is the first structure to be affected by *α*-solanine treatment [3] and indicate processes typically observed in cells exposed to metabolic or therapeutic stress [39]. In addition to inhibition of acetylcholine esterase [4], a protein containing a domain associated with cell adhesion [5], *α*-solanine also significantly interferes with the structure and function of the cell membrane [4, 6]. Progressive damage and destabilization of the mammalian cell membrane can cause cellular contents to escape [7], although the mechanisms underlying the *α*-solanine-induced changes in the barrier function of the membrane remain unclear [8].

Extensions or elongations of reticular fibrils were evident after exposure to *α*-solanine, leading to the formation of spicules. These changes are presumably related to the move towards increased adherence and a consequence of cell responses to paracrine signals, a mechanism responsible for induction of tissue repair in various types of mesenchymal cells within their morphogenetic repertoire of cell differentiation [40].

In contrast to our findings, *α*-solanine had an inhibitory effect on the growth of fibroblasts, arresting them in the G2 phase of the cell cycle [38]. A similar effect was described in HepG2 cells, where, during incremental exposure to *α*-solanine to determine IC50 (half-maximal inhibitory concentration), the proportion of cells in S phase increased, leading to a greater sensitivity of cells in G2 phase to the toxicity of this substance [41], an effect probably mediated by the Bcl-2 family of proteins which regulate apoptosis [42]. Also, the changes in the morphology of the cells may also be mediated by other mechanisms, such as suppression of the Akt/mTOR pathway and induction of autophagy [43], since both pathways have been implicated in the inhibitory activity of *α*-solanine towards tumor cells [29, 44].

Differences were also observed in the size and number of nucleoli per cell. Under normal conditions, cells have one or two distinct compact nucleoli; however, this number increases in cells requiring increased protein synthesis. Moreover, the nucleolus has roles in other cell functions, such as cell cycle regulation, stress responses, telomerase activity, and aging, and changes to this structure indicate alterations in the functional state of the cell. The results of the present study demonstrate that treatment with higher concentrations of *α*-solanine resulted in an increase in the number of nucleoli. This result is as expected, since treatment with this substance causes increased cellular stress, generating elevated protein synthesis and, therefore, a higher number of nucleoli, which have a primary function of ribosome synthesis. These changes could also be the result of the phenomenon of nuclear segregation, which is induced by various substances of vegetable origin and produces similar characteristics and effects to those observed on treatment with *α*-solanine [45].

This study found a marked difference in the percentage of adherent cells between the control group and cells exposed to 2 and 6 *μ*M of *α*-solanine; however, no such difference was observed between treatments with 2 and 4 *μ*M or 4 to 6 *μ*M *α*-solanine. These results may be attributable to a saturation effect, where the maximum influence on cell adhesion capacity was reached at a particular *α*-solanine treatment concentration, after which cell death was triggered.

Overall, our results demonstrate that *α*-solanine produces structural changes in MSCs in a dose-dependent manner, consistent with findings in other cell types, such as cardiac, colon (HT29, T84), and liver (HepG2) cells [18, 21, 46].

This study of the morphological parameters of live MSCs in adherent culture describes a simple and reproducible way to qualitatively evaluate the toxicity of glycoalkaloid treatment of multipotential cells. The study demonstrates the importance of basic qualitative observation in the understanding of biological responses of individual cells exposed to stimuli, such as glycoalkaloids [47].

## 5. Conclusions

Immunophenotypic characterization and staining of MSCs are proposed as reliable and reproducible methods that allow description of cells exposed to the glycoalkaloid, *α*-solanine. The method presented permits analysis of parameters including spicule formation, cytoplasmic condensation, the number and characteristics of nucleoli, and cell adhesion characteristics. This study highlights the importance of observations of individual cells and changes to them. The glycoalkaloid, *α*-solanine, has dose-dependent toxic effects, modifying the shape of cells, decreasing their size, increasing the number of nucleoli per cell, causing detachment of adherent cells, and decreasing intercellular adhesion. Although the study is novel, it has limitations, since it is a phenotypic description of cells in culture, which is compatible with previous studies. However, the biological basis of these changes is yet to be described. These imply future studies in relation to the analysis of apoptosis pathways in mesenchymal stem cells [41] and regulation of genes that have been related to NTD as SHROOM3 and can be sensitive to the action of glycoalkaloids during critic periods of embryogenesis [48, 49] and the study of structural changes of the cytoskeleton induced by different concentrations of *α*-solanine [50].

## Supplementary Material

Changes in proportions of adherent cells in culture.

## Figures and Tables

**Table 1:** Differences in the number of cells per colony. As the concentration of *α*-solanine increases the number of adherent's cells and the number of colonies decrease. Cell counting is based on 100 cells. (See supplementary material regarding repeated experiment results.)

Concentrations of *α*-solanine	% adherent cells	Number of colonies	Cells per colony (average)
0 *µ*M	97%	16	5.3
2 *µ*M	39%	10	4
4 *µ*M	30%	10	2.7
6 *µ*M	0%	0	0
