# Supplementary material for: A Reliable and Reproducible Model for Assessing the Effect of Different Concentrations of α-Solanine on Rat Bone Marrow Mesenchymal Stem Cells
Source: Bone Marrow Res. 2017 Oct 22;2017:2170306. doi: 10.1155/2017/2170306 (PMC5671669; doi:10.1155/2017/2170306)
Supplement: Supplementary file 1 — Changes in proportions of adherent cells in culture. [file 2170306.f1.docx]

Proportions of adherent cells in culture. No significant differences with repeated experiment (Two-sample test of proportions).

| Concentrations of α-solanine | % adherent cells | *p* | % nonadherent cells |
| --- | --- | --- | --- |
| A:Sample 1: 0 µM | 97% | 0.4 | 3% |
| A*:Sample 1: 0 µM | 95% |  | 5% |
| B: Sample 2: 2 µM | 39% | 0.6 | 61.% |
| B*: Sample 2: 2 µM | 42% |  | 58.% |
| C: Sample 3: 4 µM | 30% | 0.7 | 70% |
| C*: Sample 3: 4 µM | 32% |  | 68% |
| D: Sample 4: 6 µM | 0% | - | 100% |
| D*: Sample 4: 6 µM | 0% |  | 100% |

Cells per colony

| Concentrations of α-solanine | % adherent cells | Number of colonies | Cells per colony (average) |
| --- | --- | --- | --- |
| A:Sample 1: 0 µM | 97% | 16 | 5.3 |
| A*:Sample 1: 0 µM | 95 | 19 | 6 |
| B: Sample 2: 2 µM | 39% | 10 | 4 |
| B*: Sample 2: 2 µM | 42% | 9 | 4 |
| C: Sample 3: 4 µM | 30% | 10 | 2.7 |
| C*: Sample 3: 4 µM | 33% | 6 | 2 |
| D: Sample 4: 6 µM | 0% | 0 | 0 |
| D*: Sample 4: 6 µM | 0% | 0 | 0 |

| Marascuilo procedure: adherent cells / nonadherent cells | | | |
| --- | --- | --- | --- |
| Contrast | Value | Critical values | Significative difference |
| \|p^*^(Sample1) - p(sample 2)\| | 0,580 | 0,144 | yes |
| \|p(Sample 1) - p(Sample 3)\| | 0,670 | 0,137 | yes |
| \|p(Sample 1) - p(Sample 4)\| | 0,970 | 0,048 | yes |
| \|p(Sample 2) - p(Sample 3)\| | 0,090 | 0,187 | No |
| \|p(Sample 2) - p(Sample 4)\| | 0,390 | 0,136 | yes |
| \|p(Sample 3) - p(Sample 4)\| | 0,300 | 0,128 | yes |

^*^p=proportion

H_0_: the proportions across samples are equal.

Ha: at least one proportion in different than the others.

Interpretation: given that the p-value is less than the alfa =0 ,05, null hypothesis is rejected.

Complementary data on α - solanine

| Doses | Reference |
| --- | --- |
| Lethal dose **50** of **solanine** for mice was found to be 32·3 mg/kg. | Patil, B. C., Sharma, R. P., Salunkhe, D. K., & Salunkhe, K. (1972). Evaluation of solanine toxicity. *Food and cosmetics toxicology*, *10*(3), 395-398. |
| IC_50_ for HepG_2_, SGC-7901, and LS-174 were 14.47, >50, and >50 μg/ml | Ji, Y. B., Gao, S. Y., Ji, C. F., & Zou, X. (2008). Induction of apoptosis in HepG 2 cells by solanine and Bcl-2 protein. *Journal of Ethnopharmacology*, *115*(2), 194-202. |

Cytotoxic agent: Alpha-Solanine (Solatunin; α-Solanine), (C45H73NO15). Glycosalide trisaccharide consisting of glucose, galactose, rhamnose, and a solanidine ring, was acquired commercially. Α-Solanine is insoluble in water, chloroform and ether, therefore, was solubilized using 0.45% NaCl, 0.25% CH3COOH, and 0.2% DMSO (Dimethylsulfoxide).
